# Participant perceptions of the impact of the ModPad program on molecular diagnostic capacity building and public health practices

**DOI:** 10.3389/fpubh.2026.1799595

**Published:** 2026-04-20

**Authors:** Feng Ning, Ma Xuejun, Li Jiandong, Wang Huanyu, Wang Yufei, Zhang Yong, Dong Xiaoping

**Affiliations:** 1 Office of NCD and Ageing Health Management, Chinese Center for Disease Control and Prevention, Beijing, China; 2 National Institute for Viral Disease Control and Prevention, Chinese Center for Disease Control and Prevention, Beijing, China; 3 Women Health Department, National Center for Women and Children’s Health, National Health Commission, Beijing, China

**Keywords:** capacity building, global health, molecular diagnostics, pathogen determination, public health practices

## Abstract

**Objective:**

To investigate the perceptions of the participants having attended the International Training Program on Molecular Diagnosis and Pathogen Determination (ModPad) about its enhancing effect on their molecular diagnostic capacity as well as related public health practices.

**Method:**

A cross-sectional survey was administered to ModPad participants (both international and Chinese) using a structured questionnaire to understand their improvement in molecular techniques, knowledge transferability, satisfaction with training components, and perceived enhancements in public health service delivery and policy implementation after attending ModPad. Quantitative data were analyzed using descriptive statistics and the Chi-square test, with Fisher’s exact test applied the expected cell frequency <5. The significance threshold was set at *α* = 0.05. Qualitative responses underwent thematic analysis and word-pair frequency evaluation to extract key patterns and insights.

**Result:**

Through attending the ModPad, the top 3 technologies with the highest mastery rate reported by the international participants were PCR (87.9%), sample collection, storage and transportation (72.7%), and RNA extraction (69.7%), whereas Quantitative real-time PCR (59.5%), Next-generation sequencing (NGS, 59.5%) and PCR (56.8%) were reported by Chinese participants. Both international and Chinese participants reported challenges in acquiring expertise in complex techniques such as CRISPR-based diagnostics and third-generation sequencing (TGS). ModPad was widely recognized in the participants for its contribution to strengthening laboratory capacity and related public health services; however, its direct impact on policy development was perceived as moderate. While participants expressed high satisfaction with the program’s practical, hands-on training and in-depth technical instruction, variations in satisfaction levels emerged, particularly concerning curriculum structure and organization. Key recommendations for program improvement from the participants included further adaptability to regional needs, incorporating more advanced molecular techniques, and reinforcing policy-oriented training modules.

**Conclusion:**

The participants of the ModPad program perceived significant strengthening of their molecular diagnostic competencies, as well as the program’s role in facilitating effective knowledge dissemination among public health professionals, leading to enhanced laboratory capacity and improved service delivery.

## 1 Introduction

The rapid advancement of molecular diagnostic technique has revolutionized the detection, monitoring, and control of infectious diseases, particularly in the context of emerging and re-emerging pathogens (1–9). High precision and cutting-edge molecular techniques such as polymerase chain reaction (PCR), quantitative real-time PCR (qPCR), next-generation sequencing (NGS), and CRISPR-based diagnostics provide unparalleled sensitivity, specificity, rapid and highly specific pathogen identification (10–12). These advancements are crucial for early disease intervention, outbreak management, and global health efforts.

Simultaneously, disseminating these valuable techniques to enhance proficiency and operational capacity is critically important, particularly in low- and middle-income countries (LMICs). In these regions, constrained laboratory infrastructure, a shortage of skilled professionals, and insufficient integration of molecular techniques into public health systems collectively impede effective disease surveillance and control (13, 14). Addressing these challenges through structured, high-quality workforce training programs can significantly improve global preparedness and strengthen the resilience of public health systems.

To mitigate these challenges and bridge the gaps, the Chinese Center for Disease Control and Prevention (China CDC), created and launched the International Training Program on Molecular Diagnosis and Pathogen Determination (ModPad) in 2018. The program builds on field investigations conducted in Uganda, Gabon, and Congo (unpublished documents), incorporating real-world insights from disease surveillance and outbreak response. Unlike conventional short-term technical workshops, ModPad employed a comprehensive, structured training model that integrates hands-on laboratory instruction with leadership development in molecular diagnostics. A key focus is on capacity building among professionals from LMICs, equipping them with both technical expertise and the ability to scale up molecular diagnostics within their national public health systems. Through this approach, ModPad fostered sustainable expertise, strengthened laboratory networks, and prioritize global health practices.

Since its launch, the program has been implemented across China, Nigeria, Pakistan, and Senegal, successfully concluding 10 training cycles (November 2018–November 2021) and equipping professionals from multiple developing countries in Africa, Southeast Asia, and South Asia with advanced molecular diagnostic knowledge and skills. The curriculum encompasses core molecular techniques, including singleplex and multiplex PCR, digital PCR, isothermal amplification (RAA), and bioinformatics, ensuring a comprehensive skill set for pathogen detection and analysis. Recognizing the need for sustained capacity-building, the program expanded into ModPad+, an extended format offering on-the-job training and research collaboration at China CDC. In 2020, the program was further enhanced through the integration of molecular vaccinology, broadening its scope under the revised framework ModPad-MoV. In addition to these advancements, a systematic evaluation of the ModPad’s impact on skills acquisition, knowledge transfer, and its broader influence on public health practices was conducted in 2022. Understanding the effectiveness of molecular diagnostic training programs is essential to ensure their alignment with global public health needs, particularly in regions facing a high burden of infectious diseases.

In order to understand the perspectives and views of participants from multiple developing countries with diverse cross-cultural backgrounds on our innovative initiative in designing and implementing the ModPad (generally refers to ModPad/ModPad+/ModPad-MoV in this article), we conducted a questionnaire survey among the participants. The study aimed to investigate the ModPad’s role in enhancing participants’ mastery and transferability of molecular techniques, its contributions to public health practices, and participant satisfaction. These insights were sought to identify opportunities for improving the ModPad’s visibility and impact. Additionally, the survey analyzed how effectively participants integrated newly acquired skills into their respective health systems, providing valuable data to inform strategies for optimizing molecular diagnostic capacity building. Furthermore, the study explored the ModPad’s role in fostering international collaboration, offering critical insights for refining training methodologies and strengthening global partnerships.

## 2 Materials and methods

### 2.1 Study design

This study utilized a cross-sectional design, employing a self-administered questionnaire that comprised both closed- and open-ended questions in English. A total population sampling strategy was implemented, targeting all past international and Chinese participants of the ModPad program. The questionnaire was distributed via email in February 2022 to all participants (*n* = 185), and responses were collected electronically over a 3-week period. Out of 171 (92.4%) successfully delivered questionnaires, 70 (40.9%) responses were received (37.8% of 185 participants). The respondents included 33 international and 37 Chinese participants who voluntarily participated in the survey. Each cycle of the ModPad has respondents to this questionnaire survey who submitted their answer voluntarily. So, the time elapsed between program completion and survey participation ranged from 3 to 39 months.

In alignment with the survey objectives, the questionnaire was structured into five key dimensions, focusing on: (i) Participants’ mastery of molecular techniques and professional fields covered: This investigated their understanding of the techniques and professional fields covered in the ModPad program, along with their perceived level of difficulty. (ii) Transferability and effectiveness of acquired techniques: This investigated how well participants could apply their newly gained knowledge in their respective work contexts. (iii) Program’s role in enhancing professional capacity and epidemic response: This investigated the initiative’s contribution to strengthening participants’ skills and their ability to respond to public health emergencies. (iv) Impact on public health practices: This explored participants’ perceptions of how the acquired knowledge and skills influenced service delivery (including quantity and quality) and policy development. (v) Participant satisfaction: This captured feedback on the program’s most valuable aspects and areas for improvement. The questionnaire design included: (a) Multiple-choice options for quantitative questions about mastery and transferability of techniques; (b) 5-point Likert scale items for questions assessing the necessity, satisfaction, and importance perceived by participants; (c) Binary options (Yes/No) for questions about participants’ perceptions of the program’s improvement effects.

Prior to administering the survey, participants received a comprehensive explanation of the study’s objectives and procedures. Informed consent was obtained electronically via email, ensuring compliance with ethical guidelines. At the outset of the questionnaire, respondents were explicitly informed that: Their responses would be treated with strict confidentiality; no personally identifiable information would be disclosed; and the data would be aggregated for group-level analysis only. Participation was voluntary entirely, and respondents retained the right to withdraw at any stage without penalty. Non-participants were encouraged to discontinue the survey if they declined involvement. Submission of completed responses constituted implicit consent for inclusion in the study.

### 2.2 General setting of the ModPad

#### 2.2.1 The main contents

The core curriculum of the ModPad program encompasses a comprehensive range of topics tailored to enhance participants’ technical proficiency in molecular diagnostics and pathogen identification. The training emphasized both the foundational principles and practical applications of critical molecular techniques, including: Singleplex and multiplex polymerase chain reaction (PCR), Quantitative real-time PCR (qPCR), Digital PCR, and Nucleic acid isothermal amplification methods such as recombinase-aided amplification (RAA). Beyond these core techniques, the program introduced participants to cutting-edge technologies, including point-of-care testing (POCT), next-generation sequencing (NGS), and third-generation sequencing (TGS). Additionally, training in bioinformatics and the prevention and control of emerging and re-emerging viral infectious diseases strengthened participants’ analytical competencies, equipping them with the skills to interpret sequencing data effectively and apply molecular diagnostics in disease surveillance and outbreak response scenarios.

A distinctive feature of the ModPad program is its emphasis on leadership development, preparing participants to scale up molecular diagnostic and pathogen determination techniques within their national health systems. Recognizing the need for continued capacity building, the program evolved into ModPad+, a 12-week, on-the-job training initiative designed to bridge the gap between formal training and practical implementation in real-world settings. This extended program fostered direct connections between training activities and ongoing local research, clinical services, interventions, and public health projects. During this phase, invited participants engaged in applied research at China CDC’s research centers and departments, working on projects that align with their home countries’ research priorities. Through access to state-of-the-art technical facilities and mentorship from leading molecular diagnostic experts, participants gained hands-on experience in advanced laboratory techniques, data analysis, and translational research enhancing their ability to drive innovation in global health.

In 2020, the ModPad program expanded its scope to include molecular vaccinology, reflecting the growing global emphasis on vaccine development and deployment in infectious disease prevention. This enhancement led to the rebranding of the program as ModPad-MoV, with “MoV” signifying molecular vaccinology. The updated curriculum integrated molecular diagnostic methodologies with vaccine research and development, equipping participants with a comprehensive foundation to address infectious disease challenges through both a diagnostic and immunological lenses. By incorporating molecular vaccinology, the program strengthened its ability to provide participants with cutting-edge tools and knowledge, empowering them to contribute to global health practices.

#### 2.2.2 The technical design of ModPad

**Figure 1:**
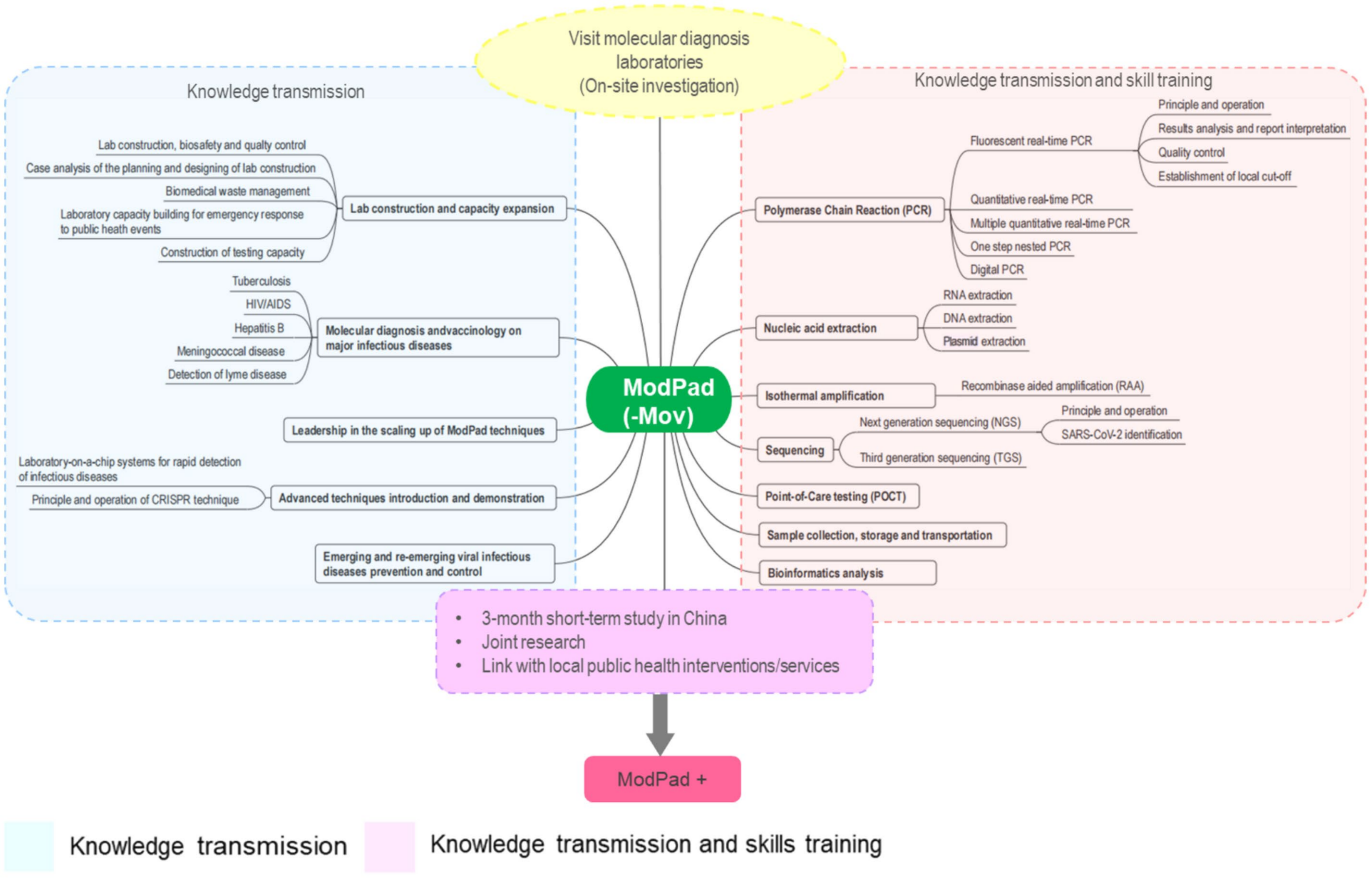
The technical design of ModPad.

The workflow of the ModPad program, highlighting the key stages in training, from knowledge transmission to skill development, is illustrated in Figure 1.

ModPad adopted module design, integrating three key components as centralized theoretical lectures, hands-on laboratory training and on-site investigations. It prioritized practical and hands-on competencies to ensure that professionals could apply theoretical knowledge in real-world settings effectively. Accordingly, ModPad framework offered a comprehensive approach to molecular diagnostics, balancing knowledge transmission with skill development.

ModPad covered essential topics such as lab construction, biosafety, quality control, biomedical waste management, and laboratory capacity building for emergency responses to infectious diseases like tuberculosis, HIV/AIDS, Hepatitis B, meningococcal disease, and Lyme disease. Additionally, the framework emphasized leadership in scaling up ModPad techniques and integrating advanced diagnostic technologies, including CRISPR, lab-on-a-chip systems, and strategies for emerging viral diseases control. The molecular diagnostic techniques trained included PCR variants (fluorescent real-time, quantitative, digital, and one-step nested PCR), nucleic acid extraction (RNA, DNA, plasmids), isothermal amplification (RAA), and next- and third-generation sequencing technologies. It also includes training in point-of-care testing (POCT), sample handling, and bioinformatics.

A defining feature of the ModPad program was its strong emphasis on international collaboration, demonstrated through a three-month short-term study in China designed for joint research and active engagement with public health initiatives. This immersive experience enabled participants to learn from global experts and apply their acquired skills in diverse healthcare environments. By bridging cutting-edge diagnostic technologies with real-world public health interventions, the ModPad ensured that advancements in pathogen detection were translated into actionable solutions effectively for disease surveillance and outbreak response.

#### 2.2.3 Organization and implementation

The ModPad program featured a structured approach to both participant and teacher invitations. For the ModPad program conducted within China, each cycle typically invited 15 to 20 international participants, along with 15 Chinese participants. For cycles held outside China (Nigeria, Pakistan, Senegal), participation was limited to 15 to 20 international attendees. The ModPad+ invited 2 to 5 international participants only per cycle. Eligible participants are carefully selected by an evaluation panel from China CDC, with selection criteria focused on professional backgrounds with senior qualification, particularly favoring those with experience in frontline pathogen testing. Chinese candidates underwent a brief telephone interview as part of the selection process. International participants represented approximately 30 developing or low-income countries in Africa, Southeast Asia, and South Asia, while Chinese participants were drawn from both national and provincial levels of Centers for Disease Control and Prevention (CDCs). Leading experts in molecular diagnostics, including top specialists from China CDC, Tsinghua University of China, the Chinese Academy of Sciences, and China’s National Gene bank, were invited to serve as instructors. Participants who successfully completed the experiments received an official certification from China CDC, recognizing their achievements in molecular diagnostics and pathogen determination.

### 2.3 Data analysis

The responses to the selective (closed-ended) questions were analyzed using descriptive statistics primarily. Participants who did not respond to a given item were excluded from that item’s analysis and denominators for each item were reported. Categorical variables were summarized using frequencies and percentages. Chi-square test (or Fisher’s exact test when the expected cell frequency <5) was applied to compare ratios between international and Chinese participants on the same indicators to investigate the effect of the ModPad objectively as well as obtain clues on how to improve the programme further. A significance level of *α* = 0.05 was used for all statistical comparisons. Software SPSS 18.0 was used for qualitative analysis.

Although both international and Chinese participants were invited to answer the open-ended questions, only the responses from international participants were reported and analyzed in this study because of its higher attractiveness and reference value. Content analysis and word pair (bigram) frequency analysis were used. A Python code, developed by the research team, was used to clean the text data, filter out stop words, and perform bigram frequency analysis. The resulting frequent word pairs were visualized in a word cloud, where the size of each phrase corresponded to its relative frequency in participant responses.

## 3 Result

### 3.1 Quantitative analysis

#### 3.1.1 Mastery and transferability of techniques

Among international participants, the three molecular techniques with the highest reported mastery rates were polymerase chain reaction (PCR) (87.9%), sample collection, storage, and transportation (72.7%), and RNA extraction (69.7%). Chinese participants most frequently mastered qPCR (59.5%), next-generation sequencing (NGS) (59.5%), and PCR (56.8%). Regarding field-specific expertise, international participants most commonly reported mastery in laboratory construction (69.7%), emerging and re-emerging viral infectious diseases (63.6%), and leadership in scaling up molecular techniques (60.6%). Chinese participants demonstrated the highest mastery rates in laboratory construction (67.6%), laboratory-on-a-chip systems (48.7%), and leadership in scaling up molecular techniques (46.0%). Statistical analysis revealed that international participants had significantly higher mastering ratios for PCR and RNA extraction than Chinese participants did (P_PCR_ = 0.0033, *χ*^2^_RNA_ = 4.0179, *p* < 0.05). Chinese participants had significantly higher mastering ratios for one step nested PCR, digital PCR, TGS and bioinformatics analysis (*χ*^2^_Nested_ = 8.8716, P _Digital_ = 0.0134, P_TGS_ = 0.0011, *χ*^2^_Bioinformatics_ = 4.7362, *p* < 0.05). International participants had significantly higher mastering ratios for field of “emerging and re-emerging viral infectious diseases prevention and control” than Chinese participants (*χ*^2^ = 6.8158, *p* < 0.05). The most frequently transferred techniques and fields by international participants to their home institutions aligned with the three most commonly mastered techniques (Table 1).

**Table 1:** Participants’ mastering and transferring status of techniques and fields in ModPad (*n*, %).

Techniques and fields	INT P. (*n* = 33)	CHN P. (*n* = 37)	Subtotal (*n* = 70)	*p*-value (*χ*^2^ value)	Transfer by INT P.
Techniques					
PCR	29, 87.9	21, 56.8*^#^	50, 71.4	0.0033^	21, 63.6
qPCR	17, 51.5	22, 59.5	39, 55.7	>0.05 (0.4461)	10, 30.3
Multiple qPCR	11, 33.3	15, 40.5	26, 37.1	>0.05 (0.3881)	6, 18.2
One step nested PCR	5, 15.2	18, 48.7*	23, 32.9	<0.05 (8.8716)	2, 6.1
Fluorescent real-time PCR	9, 27.3	17, 46.0	26, 37.1	>0.05 (2.6051)	3, 9.1
RNA extraction	23, 69.7	17, 46.0*	40, 57.1	<0.05 (4.0179)	16, 48.5
RAA	6, 18.2	13, 35.1	19, 27.1	0.0622^	2, 6.1
Digital PCR	5, 15.2	15, 40.5*^#^	20, 28.6	0.0134^	0, 0.0
POCT	14, 42.4	13, 35.1	27, 38.6	>0.05 (0.3911)	8, 24.2
CRISPR	3, 9.1	7, 18.9	10, 14.3	0.1416^	0, 0.0
NGS	14, 42.4	22, 59.5	36, 51.4	>0.05 (2.0264)	9, 27.3
TGS	3, 9.1	16, 43.2*^#^	19, 27.1	0.0011^	2, 6.1
Sample collection, storage and transportation	24, 72.7	19, 51.4	43, 61.4	>0.05 (3.3638)	13, 39.4
Bioinformatics analysis	7, 21.2	17, 46.0*	24, 34.3	<0.05 (4.7362)	5, 15.2
Field					
Lab construction, biosafety, and quality control	23, 69.7	25, 67.6	48, 68.6	0.1997^	19, 57.6
Molecular diagnosis and molecular vaccinology of Hepatitis B	9, 27.3	9, 24.3	18, 25.7	0.2068^	4, 12.1
Molecular diagnosis and prevention of tuberculosis	9, 27.3	9, 24.3	18, 25.7	0.2068^	3, 9.1
Molecular diagnosis and molecular vaccinology of HIV/AIDS	9, 27.3	11, 29.7	20, 28.6	0.2037^	1, 3.0
Laboratory-on-a-chip systems for rapid detection of infectious diseases	10, 30.3	18, 48.7	28, 40.0	>0.05 (2.4461)	1, 3.0
Emerging and re-emerging viral infectious diseases prevention and control	21, 63.6	12, 32.4*	33, 47.1	<0.05 (6.8158)	10, 30.3
Leadership in the scaling up of molecular diagnosis and pathogen determination techniques	20, 60.6	17, 46.0	37, 52.9	>0.05 (1.5044)	12, 36.4

INT P., international participants; CHN P., Chinese participants.

^*^p < 0.05, INT P. vs. CHN P. ^**#**^Fisher’s exact test was used for comparison.

^The *p*-value was calculated through Fisher’s exact test.

The three highest reported difficult mastering techniques were TGS (51.5%), bioinformatics analysis (48.5%), and CRISPR (42.4%) among international participants, and bioinformatics analysis (54.1%), CRISPR (48.7%) and TGS (37.8%) among Chinese participants. International participants had significantly higher ratios of difficult mastering techniques for one step nested PCR and RAA as well as fields for “molecular diagnosis and molecular vaccinology of Hepatitis B” and “molecular diagnosis and molecular vaccinology of HIV/AIDS” for than Chinese participants did, respectively (P_Nested_ = 0.0196, P_RAA_ = 0.0048, P_Hepatitis_ = 0.0079, P_HIV_ = 0.0499) (Table 2).

**Table 2:** The participants’ difficult mastering status of techniques and fields in ModPad (*n*, %).

Techniques and fields	INT P. (*n* = 33)	CHN P. (*n* = 37)	Subtotal (*n* = 70)	*p*-value (*χ*^2^ value)
Techniques				
PCR	1, 3.0	0, 0.0	1, 1.4	0.4714^
qPCR	3, 9.1	0, 0.0	3, 4.3	0.0997^
Multiple qPCR	5, 15.2	1, 2.7	6, 8.6	0.067^
One step nested PCR	5, 15.2	0, 0.0*^#^	5, 7.1	0.0196^
Fluorescent real-time PCR	2, 6.1	0, 0.0	2, 2.9	0.2186^
RNA extraction	1, 3.0	0, 0.0	1, 1.4	0.4714^
RAA	13, 39.4	4, 10.8*^#^	17, 24.3	0.0048^
Digital PCR	8, 24.2	5, 13.5	13, 18.6	0.1275^
POCT	7, 21.2	5, 13.5	12, 17.1	0.175^
CRISPR	14, 42.4	18, 48.7	32, 45.7	>0.05 (0.2723)
NGS	10, 30.3	10, 27.0	20, 28.6	0.1992
TGS	17, 51.5	14, 37.8	31, 44.2	>0.05 (0.3225)
Sample collection, storage and transportation	0, 0.0	1, 2.7	1, 1.4	>0.05 (1.3225)
Bioinformatics analysis	16, 48.5	20, 54.1	36, 51.4	>0.05 (0.2166)
Field				
Lab construction, biosafety, and quality control	1, 3.0	5, 13.5	6, 8.6	0.1097^
Molecular diagnosis and molecular vaccinology of Hepatitis B	8, 24.2	1, 2.7*^#^	9, 12.9	0.0079^
Molecular diagnosis and prevention of tuberculosis	5, 15.2	2, 5.4	7, 10.0	0.1319^
Molecular diagnosis and molecular vaccinology of HIV/AIDS	8, 24.2	3, 8.1*^#^	11, 15.7	0.0499^
Laboratory-on-a-chip systems for rapid detection of infectious diseases	6, 18.2	7, 18.9	13, 18.6	0.2402^
Emerging and re-emerging viral infectious diseases prevention and control	3, 9.09	5, 13.5	8, 11.4	0.2519^
Leadership in the scaling up of molecular diagnosis and pathogen determination techniques	4, 12.12	7, 18.9	11, 15.7	0.1947^

INT P., international participants; CHN P., Chinese participants.

*p < 0.05, INT P. vs. CHN P. ^**#**^Fisher’s exact test was used for comparison.

^The *p*-value was calculated through Fisher’s exact test.

#### 3.1.2 Necessity, satisfaction and capacity improvement

**Figure 2:**
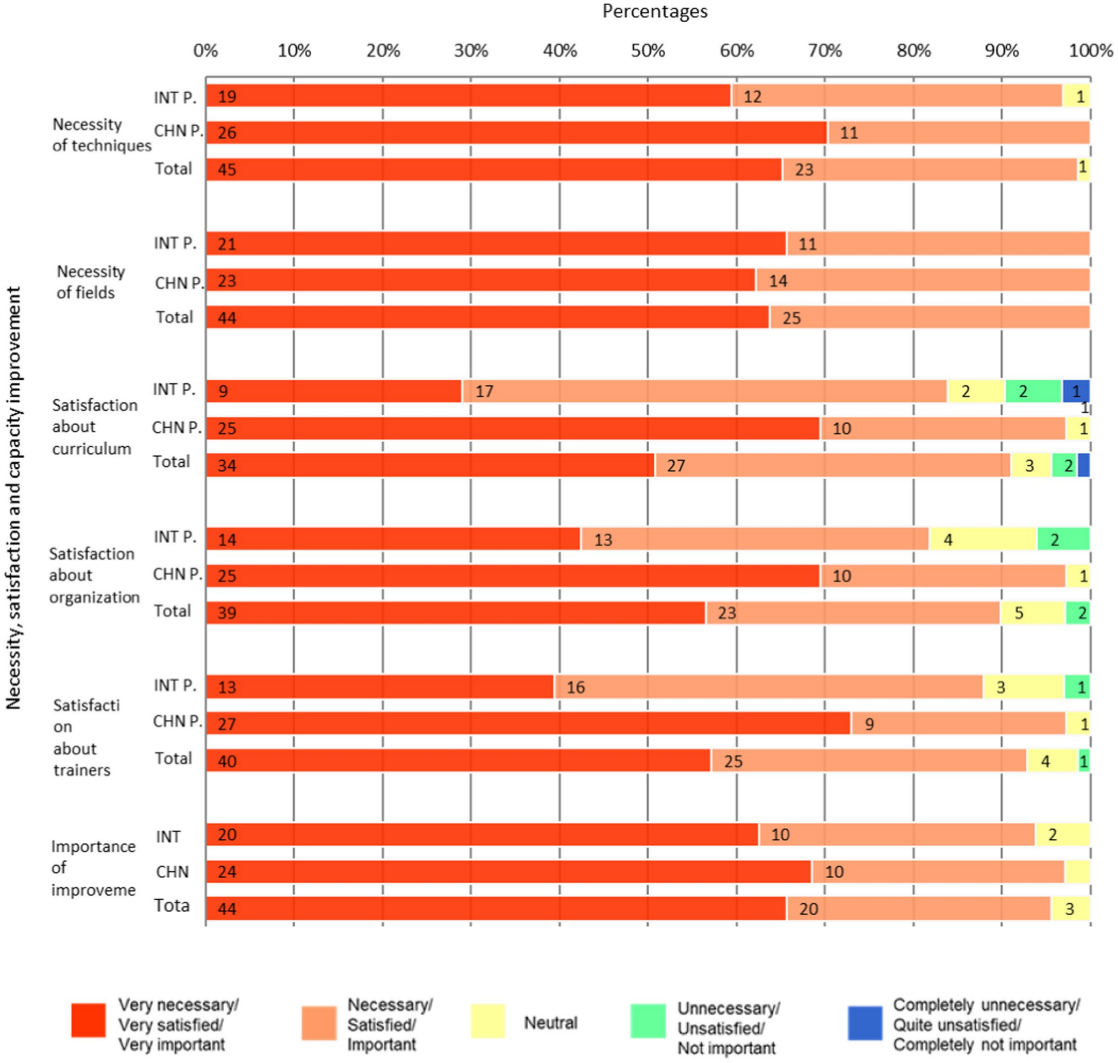
Necessity, satisfaction, and importance perceived by the participants of the ModPad.

Among surveyed participants, 57.6% of international respondents and 70.3% of Chinese respondents rated the techniques taught in the ModPad as “very necessary.” When combining the responses categorized as “very necessary” and “necessary,” the proportions increased to 93.9% for international participants and 100% for Chinese participants. Similarly, for the fields covered in the ModPad, 63.6% of international participants and 62.2% of Chinese participants deemed them “very necessary,” with combined proportions (“very necessary” + “necessary”) reaching 100% in both groups. No statistically significant difference of “very necessary” ratio was observed between Chinese and international participants (*χ*^2^_technique_ = 1.2243, *χ*^2^_field_ = 0.0162, *p* > 0.05; Figure 2).

Regarding curriculum satisfaction, 27.3% of international participants and 67.6% of Chinese participants reported being “very satisfied.” The combined proportions of “very satisfied” and “satisfied” responses rose to 78.8% for international participants and 94.6% for Chinese participants. For program organization satisfaction, 42.4% of international participants and 67.6% Chinese participants were “very satisfied,” increasing to 81.8 and 94.6%, respectively, when including “satisfied” responses. A comparable trend emerged for trainer satisfaction, where 39.4% of international participants and 73.0% of Chinese participants expressed being “very satisfied,” with overall satisfaction rates (combining “very satisfied” and “satisfied”) reaching 87.9 and 97.3%, respectively.

Statistical analysis indicated that the proportions of participants reporting “very satisfied” responses were significantly higher among Chinese participants compared to international participants across all satisfaction domains: curriculum (*χ*^2^ = 11.3379, *p* < 0.05), program organization (*χ*^2^ = 4.4692, *p* < 0.05), and trainer quality (*χ*^2^ = 8.0310, *p* < 0.05) (Figure 2).

Regarding perceptions of capacity improvement through the ModPad, 60.6% of international participants and 64.9% of Chinese participants rated its role to be “very important” in enhancing professional capabilities. When combining the responses of “very important” and “important,” the proportions rose to 90.9% for international participants and 91.9% for Chinese participants. However, no statistically significant difference was observed between the two groups (*χ*^2^ = 0.7106, *p* > 0.05) (Figure 2).

#### 3.1.3 Perceived contribution to public health practices

A majority of participants reported that the knowledge and skills acquired through the ModPad contributed to improvements in public health service delivery in their home countries. Specifically, 87.9% of international participants and 83.8% of Chinese participants indicated that their training facilitated an increase in the quantity of relevant public health services, including testing, diagnosis, and research activities. Similarly, 81.8% of international participants and 72.9% of Chinese participants perceived an enhancement in the quality of these services as a direct result of their contributions post-program.

**Figure 3:**
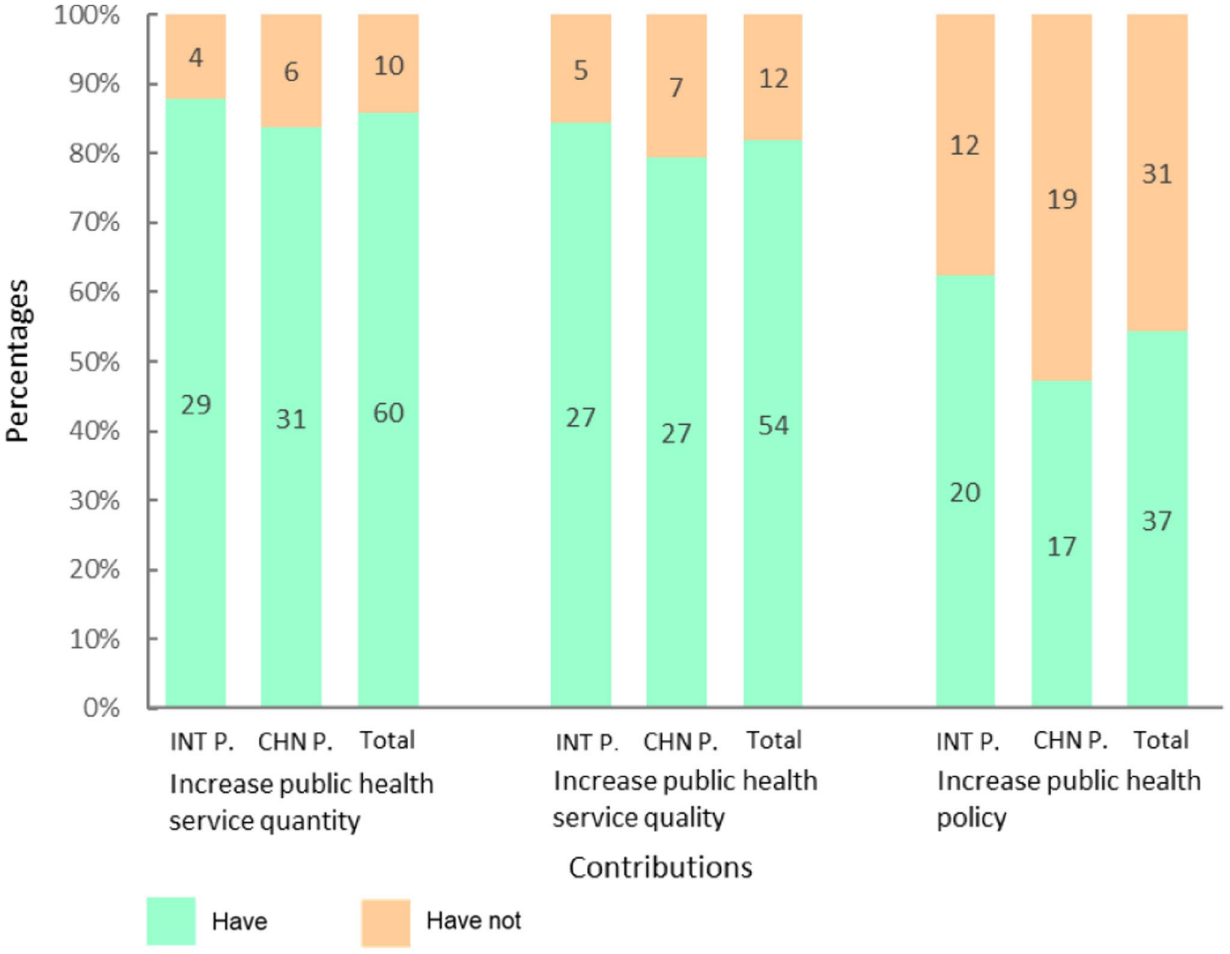
The improvement effect of the ModPad on the quantity, quality and policy of public health services.

Regarding the impact on public health policy, 60.6% of international participants and 46.0% of Chinese participants reported that the ModPad training contributed to policy advancements in their respective countries. No statistically significant difference was observed between the two groups in their perceptions of improvements in service quantity, quality, or policy impact (P_quantity_ = 0.2398, P_quality_ = 0.2201, P_policy_ = 0.0886 in Fisher’s exact test) (Figure 3).

Notably, the overall proportion of participants reporting policy improvements was significantly lower than those reporting increases in public health service quantity (χ^2^ = 16.1841, *p* < 0.05) and quality (*χ*^2^ = 11.5439, *p* < 0.05), suggesting that the ModPad had a stronger impact on technical capacity and service provision than on direct policy development (Figure 3).

### 3.2 Qualitative analysis

#### 3.2.1 Challenges encountered in the transferability of knowledge

When international participants were asked: “If you have transferred any techniques delivered in ModPad/ModPad+/ModPad-Mov training in your homeland, what challenges have you encountered,” about 2/3 cited resource limitations, including facilities, electricity, equipment, reagents, consumables, funding or financial support, and suitable samples for method validation and optimization etc. They had been even “struggling for” the resources. In addition, insufficient logistical operational capacity also brought a tangible challenge.

*“Commodity flow disruptions at testing centers during high sample volumes create operational gaps.” “The biosafety concept in application requires support to train personnel in peripheral laboratories who are involved in sample collection. This has not taken full shape because of logistical issues.”*

Sixteen of 33 (48.5%) international participants reported challenges related to techniques and knowledge weakness especially for the bioinformatics analysis after NGS. Further, the transfers of NGS and TGS were big challenges because of lack of experimental condition in their hometown. Moreover, international participants required the technical support for standard operation.

*“I need standard operating procedures (SOPs) of the experimental methods when preparing to transfer the techniques in my hometown.”*

Some participants highlighted human capacity constraints. *“Majority of the human resources are unfamiliar with molecular biology testing, requiring extensive training.”* Only one participant said *“Not a big challenge.”* And some participants express their confidence in applying techniques learnt to improve work and scientific research.

#### 3.2.2 Improvement in professional capacity

When international participants were asked: “How ModPad/ModPad+/ModPad-Mov training improved your professional capacity,” most respondents articulated their achievements in skill development vividly. They highlighted that the ModPad expanded their expertise in several key areas:

*“The training significantly deepened my understanding of result interpretation through amplification curve analysis during testing, thereby strengthening my knowledge of molecular diagnostics for* var*ious infections affecting diverse populations. Additionally, it introduced me to new POCT technologies, as well as isothermal amplification techniques like RAA, which are relevant for timely diagnosis of diseases at the point of sample collection.”*

Most participants said the ModPad has improved their skills such as PCR technique, interpretation of PCR results, sample collections, RNA extraction and Biosafety principles etc. They became aware of their earlier improper placement and cleaning of the cabinet, which could compromise user safety. Additionally, *“the participants were introduced to the bioinformatics pipeline developed by scientists in China CDC,” “I mastered the isothermal detection method and developed the first reagent in my homeland.”*

Some participants emphasized that their daily work had improved after attending the ModPad, particularly in terms of enhanced day to day work communications and implementations, knowledge transfer to students, and strengthened capacities in infectious diseases diagnostics, biosafety, infection prevention and control issues, and quality control. *“I benefited greatly from the training and started developing a plan by implementing the quality system in lab.” “The training inspired me to pay more attention to NGS and TGS. I cannot wait to lay my hands on it and become a master in sequencing.”*

#### 3.2.3 The most impressing aspects

When the international participants were asked: “What impressed you most during ModPad/ModPad+/ModPad-Mov training,” first and foremost, the training organization, facilities and supportive environment left a lasting impression.

*“The ModPad training are exceptionally well organized and meticulously planned, incorporating both theoretical lectures and practical hands-on components. The organizer worked hard and tried the best for the training. We as participants were granted access to the state-of-the-art laboratories, where we could process sample and handle highly sophisticated equipment actively. There were representatives from diverse countries given equal opportunities to participate and contribute data from their respective countries. Another important aspect that impressed me was the vast disease control and prevention system, performing at provincial and at district level throughout China. It’s a huge network of laboratories and the level of coordination and communication was phenomenal. The detection of any infectious disease in a matter of days and hours determines the success of the system.”*

Furthermore, participants praised the staff at China CDC for their friendliness and welcoming demeanor, deep technical expertise and always-on assistance, as well as the lecturer’s high level of professionalism.

*“The lectures for senior scientists working in the frontier of knowledge was very informative and inspiring.” “The trainers were very knowledgeable, lovely and willing to share their knowledge. I was also impressed by the innovative way of doing things.” “The detailed presentation and good content shared impressed me most.”*

Separately, a few of international participants considered it was technological progress.

*“The technology of vaccine production using molecular techniques, advancement in diagnostic techniques impressed me most.”*

#### 3.2.4 Word pair frequency analysis

**Figure 4:**
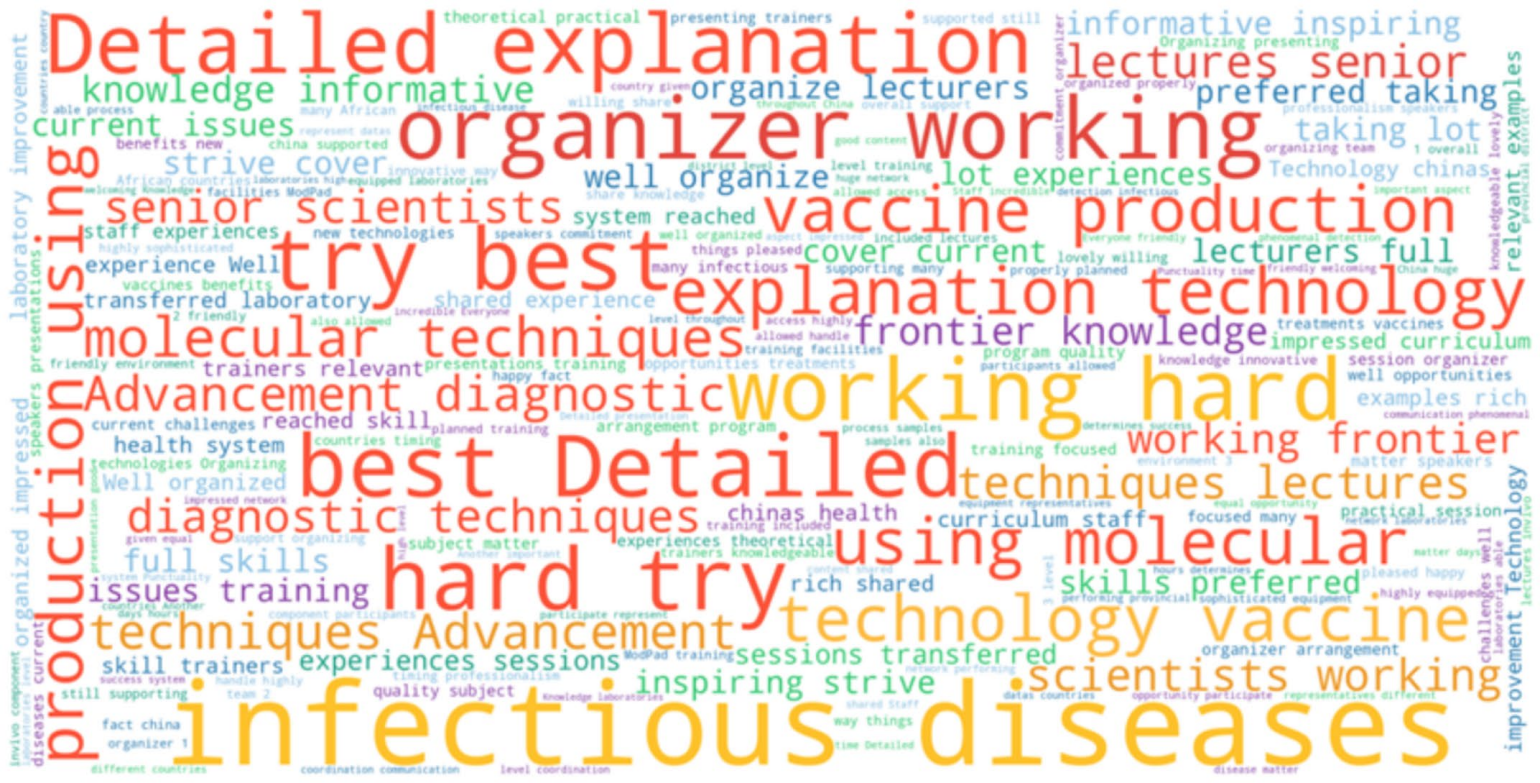
Notional word pair frequency of international participants’ most impressed point on ModPad.

A word cloud analysis was employed to showcase the most frequently mentioned aspects of ModPad that impressed international participants. The analysis emphasized several key themes prominently featured in the word cloud, including “Infectious diseases,” “organizer working,” “working hard,” “detailed explanation” and “try best” and so on (Figure 4).

## 4 Discussion

Participant feedback indicated that the ModPad had enhanced individual competencies in public health laboratory technology while expanding the scope of capacity building for public health professionals. Amidst global pandemic responses, the program has demonstrated practical significance by equipping professionals with essential molecular diagnostic expertise for pathogen detection, outbreak management, and epidemiological surveillance. High levels of participant satisfaction reported by both international and Chinese attendees further underscore the program’s perceived effectiveness in skill acquisition, knowledge transfer, and capacity strengthening.

Beyond individual skill development, participants perceived that the ModPad influenced the quantity and quality of public health services positively although the impact on policy optimization was reported as limited comparatively. As an international training platform, the ModPad has facilitated the dissemination of advanced molecular diagnostic technologies in developing and LICs, supporting global epidemic preparedness and response. This is particularly crucial for early pathogen identification, rapid diagnosis, and timely intervention in infectious disease outbreaks. Additionally, China’s extensive expertise in molecular diagnostic technology has provided unique and valuable support through technical expertise, innovative methodologies, and high-quality diagnostic products.

International participants had different home-environment, culture background, and pre-training mastery level of the molecular diagnostic techniques with Chinese ones but the two groups learnt and worked together through attending the ModPad. So, meaningful findings were obtained by comparing the two groups. The comparative analysis of technique mastery between international and Chinese participants revealed comparatively significant differences in learning outcomes. International participants exhibited higher mastery levels in fundamental molecular techniques such as PCR and RNA extraction, reflecting their widespread application in international laboratory settings and established use in participants’ home countries. Meanwhile, international participants reported greater challenges in mastering more advanced and computationally intensive techniques, including CRISPR-based diagnostics, TGS and bioinformatics analysis. These findings suggest that cutting-edge molecular methodologies require additional specialized training and infrastructure support while basic molecular diagnostic techniques are well-integrated into international laboratory training programs.

In contrast, Chinese participants demonstrated significantly higher mastery in one-step nested PCR, digital PCR, and laboratory-on-a-chip systems for rapid detection of infectious diseases. Their enhanced proficiency in these areas reflects a stronger focus and learning engagement in high-throughput and miniaturized diagnostic technologies, likely influenced by China’s research priorities and technological advancements in these domains. Another, Chinese participants trained on Chinese soil, with familiar equipment and colleagues available for follow-up questions. While, most international participants trained in an unfamiliar environment, in some cases with language barriers (English-only curriculum for multilingual participants), and returned home to environments where the equipment used in training was unavailable. These contextual factors may also the explanation for the observed mastery difference.

Both groups encountered challenges in mastering certain advanced techniques, yet noticeable differences emerged in their reported difficulty levels. CRISPR-based diagnostics, TGS, and bioinformatics analysis proved particularly arduous for both groups, demonstrating their low transferability and underscoring the necessity for specialized training and enhanced computational skill development to attain proficiency in these areas. This also suggested a programme improvement opportunity to enhance the hands-on laboratory time for CRISPR and TGS within the ModPad curriculum.

Regarding satisfaction with the program, international participants reported a significantly lower level of “very satisfied” responses than their Chinese counterparts (*p* < 0.05). However, when combining “very satisfied” and “satisfied” categories, both groups exhibited comparable overall satisfaction levels, suggesting that international participants maintained a positive perception of the ModPad albeit with stricter subjective evaluation criteria.

Qualitative feedback further supported this observation, with international participants specifically highlighting appreciation for the program’s rigor, depth, and hands-on training components. A critical theme observed from the feedback was the need for continuous improvement, reflecting participants’ recognition of the ModPad’s value and their expectations for its ongoing refinement. Both international and Chinese participants acknowledged the ModPad’s effectiveness and emphasized the necessity of enhancing its global impact through iterative improvements and curriculum updates.

Most participants acknowledged that the ModPad had contributed positively to both the quantity and quality of public health services in their home countries. A key finding was that while the ModPad successfully improved service quantity and quality, its perceived impact on policy development was significantly weaker (*p* < 0.05), suggesting the need for narrowing the gap between capacity-building initiatives and policy frameworks, particularly leveraging policies to enrich resource and optimize methodology in scaling up molecular diagnostic techniques.

Participant feedback has highlighted the structured design of the ModPad as its most valuable aspects, with many emphasizing the exceptional impact of hands-on training in enhancing their skills and confidence. However, the effective implementation of laboratory-based operational techniques depends heavily on adequate material resources, which poses a significant challenge for developing countries. Some participants reported difficulties stemming from material shortages and supply chain constraints, which hindered their ability to apply newly acquired skills in their home institutions. To address these challenges, collaborative strategies are essential to enhance resource availability, including strengthening partnerships with international organizations, promoting resource-sharing initiatives, and facilitating access to essential laboratory materials.

The word cloud analysis of qualitative responses further illuminated key themes in participant experiences. International participants consistently described the ModPad as rigorous and intellectually demanding, emphasizing its in-depth explanations and structured training approach. Notably, frequent mentions of “working hard” and “best” suggest that participants highly valued the intellectual and practical effort required to master complex topics, ultimately fostering a sense of achievement and skill enhancement upon program completion. Additionally, frequent references to “trainers” and “organizers” highlighted the critical role of expert facilitation, clear instructional delivery, and a well-designed curriculum in driving the program’s success.

To enhance the capabilities of public health professionals in addressing diverse practical needs, leaders in the field have developed several specialized training programs, including the Structured Operational Research Training Initiative (SORT IT) and the Field Epidemiology Training Program (FETP). These initiatives have demonstrated significant effectiveness in capacity-building efforts. Specifically, SORT IT focuses on strengthening health systems through operational research training (15), while FETP was designed to cultivate field epidemiology expertise, enabling professionals to effectively detect and respond to outbreaks (16–18). While, there had been a shortage of renowned training project involving public health laboratory skills particularly in advanced molecular diagnosis techniques. The importance of sustained collaboration with laboratory partners and continuous professional development in advanced laboratory technologies has been well-documented (19). In this context, the development and implementation of the ModPad program exemplified a leading practice in advancing global health strategies (20), directly addressing these critical capacity-building needs.

Actually, molecular diagnostic technology extends far beyond pathogen detection and plays an increasingly vital role in etiological (pathogeny) identification across various fields of medicine. Its applications in maternal and child health, oncology, and non-communicable chronic disease (NCD) control have demonstrated significant public health benefits. Molecular diagnostics is now widely utilized in screening and diagnosing congenital diseases, prenatal health assessments, early cancer detection, and identifying high-risk genetic and environmental factors associated with NCDs (21–25). The positive effect of the ModPad in spreading universal molecular diagnostic knowledge and skills as well as its modular design with hand-on element provided a supportive basis for the feasibility of expanding it to molecular diagnosis training for above etiology. This prospect for its future practice would transform the ModPad into a comprehensive training platform, integrating disease prevention, early detection, and control technologies, particularly tailored to address the needs of developing countries.

This study is not without limitations. The reported mastery post-course might be influenced by existing mastery prior to attending the course without rigorous entry level evaluation of skills/mastery of the techniques being taught. However, the relevant questions in the questionnaire emphasized the knowledge and skills acquired “through training.” Phone interview (Chinese applicants) or resume screening (international applicants) during participant recruitment could help identify applicants who had already mastered the relevant skills, allowing for their exclusion from enrollment to the training. These mitigated the deviation in the measuring of mastery ratio.

## 5 Conclusion

The participants of the ModPad program perceived significant strengthening of their molecular diagnostic competencies, as well as the program’s role in facilitating effective knowledge dissemination among public health professionals, leading to enhanced laboratory capacity and improved service delivery. Future iterations may prioritize targeted curriculum refinements, the integration of advanced molecular techniques, and policy-focused training modules to optimize its impact on global health and practical implementation.

## Data Availability

The original contributions presented in the study are included in the article/supplementary material, further inquiries can be directed to the corresponding authors.
